# Supplementary material for: Drug repurposing for aging research using model organisms
Source: Aging Cell. 2017 Jun 16;16(5):1006–15. doi: 10.1111/acel.12626 (PMC5595691; doi:10.1111/acel.12626)
Supplement: Supplementary file 7 — Data S1 Zip‐Archive of all report cards. [file ACEL-16-1006-s007.zip › RC_QIG.pdf]

QIG

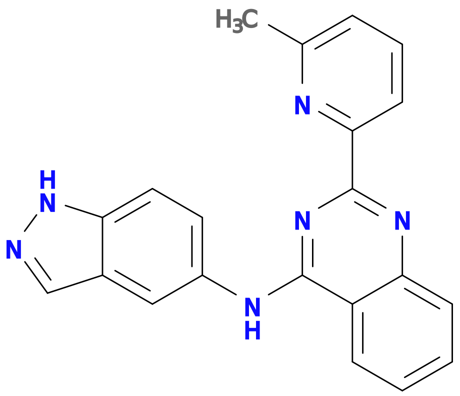

Database identifiers

ChEMBLCompound	CHEMBL537968
DrugBank	DB08450
ZINC	ZINC39279791

Ranking

	Rank	Score
Drosophila	106/697	0.785
C. elegans	NA	NA

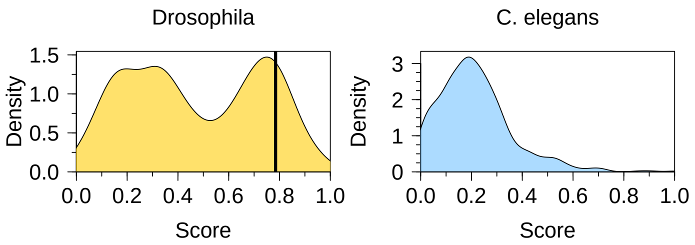

	Ageing implication	Domain conservation	Binding site conservation	Binding affinity	Bioavailability	Lipinski	Promiscuity	Purchasability	Drug approval	**Total**
Drosophila	0.792	0.958	0.966	0.924	(0.9)	0.0	-0.0	0.1	0.075	0.785
C. elegans	NA	NA	NA	NA	NA	NA	NA	NA	NA	NA

Names

No synonyms found

Roles

ChEBI entry None has no roles

Status

Approved drug (according to ChEMBL)	No
Classification (according to DrugBank)	experimental
Number of Rule of 5 violations	0
Binding affinity to original target in log units (RF-Score prediction)	7.5
Burns *C. elegans* bioavailability prediction	6.6

Compound Target Characteristics

TGF-beta receptor type-1

Best gene implication in ageing for this target family came from gene P80204 annotated in UniProt release 2014_02. Annotation GO 7568 (aging) was Inferred from Expression Pattern

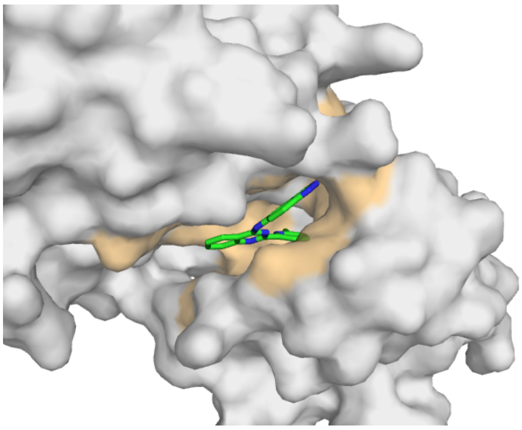

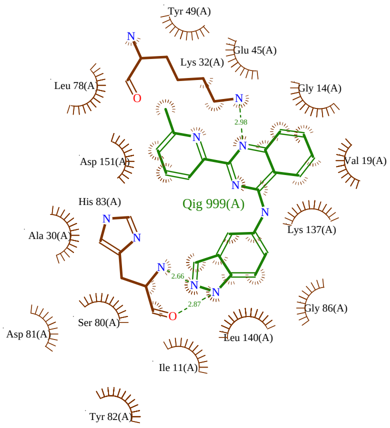

protein	amino acids contacts (binding site)
PDB:3gxl:chainA:P36897	I G V A K E Y L S D Y H G K L D
tr:F8W1R9:F8W1R9_HUMAN	I G V A K E Y L S D Y H G K L D
tr:B4DY26:B4DY26_HUMAN	I G V A K E Y L S D Y H G K L D
tr:Q5T7S2:Q5T7S2_HUMAN	I G V A K E Y L S D Y H G K L D
sp:P36897:TGFR1_HUMAN	I G V A K E Y L S D Y H G K L D
tr:Q5M9H3:Q5M9H3_RAT	I G V A K E Y L S D Y H G K L D
sp:P80204:TGFR1_RAT	I G V A K E Y L S D Y H G K L D
tr:Q3U485:Q3U485_MOUSE	I G V A K E Y L S D Y H G K L D
tr:Q9D5H8:Q9D5H8_MOUSE	I G V A K E Y L S D Y H G K L D
tr:E9Q418:E9Q418_MOUSE	I G V A K E Y L S D Y H G K L D
sp:Q64729:TGFR1_MOUSE	I G V A K E Y L S D Y H G K L D
tr:Q4FJL1:Q4FJL1_MOUSE	I G V A K E Y L S D Y H G K L D
tr:Q7YU60:Q7YU60_DROME	I G V A K E Y L T D Y H G K L D
tr:A1Z7L9:A1Z7L9_DROME	I G V A K E Y L T D Y H G K L D
tr:Q23975:Q23975_DROME	I G V A K E Y L T D Y H G K L D
tr:A1Z7L8:A1Z7L8_DROME	I G V A K E Y L T D Y H G K L D

protein	whole protein ident	simil	domain-based ident	simil	contact-based ident	simil
PDB:3gxl:chainA:P36897	1.0	1.0	1.0	1.0	1.0	1.0
tr:F8W1R9:F8W1R9_HUMAN	0.74	0.74	0.59	0.59	1.0	1.0
tr:B4DY26:B4DY26_HUMAN	0.86	0.86	1.0	1.0	1.0	1.0
tr:Q5T7S2:Q5T7S2_HUMAN	1.0	1.0	1.0	1.0	1.0	1.0
sp:P36897:TGFR1_HUMAN	1.0	1.0	1.0	1.0	1.0	1.0
tr:Q5M9H3:Q5M9H3_RAT	0.96	0.98	1.0	1.0	1.0	1.0
sp:P80204:TGFR1_RAT	0.95	0.97	1.0	1.0	1.0	1.0
tr:Q3U485:Q3U485_MOUSE	0.82	0.83	1.0	1.0	1.0	1.0
tr:Q9D5H8:Q9D5H8_MOUSE	0.77	0.8	1.0	1.0	1.0	1.0
tr:E9Q418:E9Q418_MOUSE	0.85	0.86	1.0	1.0	1.0	1.0
sp:Q64729:TGFR1_MOUSE	0.96	0.97	1.0	1.0	1.0	1.0
tr:Q4FJL1:Q4FJL1_MOUSE	0.96	0.97	1.0	1.0	1.0	1.0
tr:Q7YU60:Q7YU60_DROME	0.47	0.69	0.72	0.91	0.94	0.97
tr:A1Z7L9:A1Z7L9_DROME	0.47	0.7	0.72	0.91	0.94	0.97
tr:Q23975:Q23975_DROME	0.47	0.7	0.72	0.91	0.94	0.97
tr:A1Z7L8:A1Z7L8_DROME	0.45	0.67	0.72	0.91	0.94	0.97

babo (FBgn0011300) associated phenotypes

cell growth defective, decreased cell number, developmental rate defective, dominant, flight defective, germline clone, long lived, mitotic cell cycle defective, neuroanatomy defective, phototaxis defective, progressive, size defective, somatic clone

(Information from FlyBase)
